# In Vivo siRNA Delivery to Immunosuppressive Liver Macrophages by α-Mannosyl-Functionalized Cationic Nanohydrogel Particles

**DOI:** 10.3390/cells9081905

**Published:** 2020-08-15

**Authors:** Leonard Kaps, Nadine Leber, Adrian Klefenz, Niklas Choteschovsky, Rudolf Zentel, Lutz Nuhn, Detlef Schuppan

**Affiliations:** 1 Institute of Translational Immunology and Research Center for Immunotherapy (FZI), University Medical Center of the Johannes Gutenberg-University Mainz, Obere Zahlbacher Str. 63, 55131 Mainz, Germany; leonardkaps@googlemail.com (L.K.); aklefenz@students.uni-mainz.de (A.K.); niklas.choteschovsky@gmx.de (N.C.); 2 First Department of Medicine, University Medical Centre of the Johannes Gutenberg-University, Langenbeckstrasse 1, 55131 Mainz, Germany; 3 Department of Chemistry, Johannes Gutenberg-University of Mainz, Duesbergweg 10-14, 55128 Mainz, Germany; nadine.leber.89@gmail.com (N.L.); zentel@uni-mainz.de (R.Z.); 4 Max-Planck-Institute for Polymer Research, Ackermannweg 10, 55128 Mainz, Germany; 5 Division of Gastroenterology, Beth Israel Deaconess Medical Center, Harvard Medical School, 330 Brookline Avenue, Boston, MA 02215, USA

**Keywords:** nanohydrogels, mannose targeting, M2 macrophages, gene knock-down, siRNA delivery, fibrosis, cancer, immunotherapy

## Abstract

Macrophages are the front soldiers of the innate immune system and are vital for immune defense, tumor surveillance, and tissue homeostasis. In chronic diseases, including cancer and liver fibrosis, macrophages can be forced into an immunosuppressive and profibrotic M2 phenotype. M2-type macrophages overexpress the mannose receptor CD206. Targeting these cells via CD206 and macrophage repolarization towards an immune stimulating and antifibrotic M1 phenotype through RNA interference represents an appealing therapeutic approach. We designed nanohydrogel particles equipped with mannose residues on the surface (ManNP) that delivered siRNA more efficiently to M2 polarized macrophages compared to their untargeted counterparts (NonNP) in vitro. The ManNP were then assessed for their in vivo targeting potential in mice with experimental liver fibrosis that is characterized by increased profibrotic (and immunosuppressive) M2-type macrophages. Double-labelled siRNA-loaded ManNP carrying two different near infrared labels for siRNA and ManNP showed good biocompatibility and robust uptake in fibrotic livers as assessed by in vivo near infrared imaging. siRNA–ManNP were highly colocalized with CD206^+^ M2-type macrophages on a cellular level, while untargeted NP (NonNP) showed little colocalization and were non-specifically taken up by other liver cells. ManNP did not induce hepatic inflammation or kidney dysfunction, as demonstrated by serological analysis. In conclusion, α-mannosyl-functionalized ManNP direct NP towards M2-type macrophages in diseased livers and prevent unspecific uptake in non-target cells. ManNP are promising vehicles for siRNA and other drugs for immunomodulatory treatment of liver fibrosis and liver cancer.

## 1. Introduction

Immune therapy represents a novel type of treatment, which boosts the body’s immune system to recognize and eliminate pathogens or abnormal cells, as in cancers, and thus establish a defense memory. Therefore, immune therapy has become a central pillar in cancer treatment, either as first-line therapy or as an additive to classical chemotherapy. In the late 19th century, cancer immune therapy was initiated by Coley’s toxin, a mixture of killed streptococcus bacteria, then progressed by the use of systemic cytokines, such as interleukin (IL)-2, and evolved to the more recently approved checkpoint inhibitors anti-CTLA-4 and anti-PD-1/PD-L1 [1].

Tumor cells acquire three major features to evade immune recognition in order to grow and progress: the ability to 1. thrive in a chronically inflamed, immunosuppressive microenvironment; 2. evade immune recognition by the host; 3. actively suppress the host’s immune response [1].

The (advanced) tumor microenvironment consists of subsets of immunosuppressive cells, and macrophages often play a central in cancer immune suppression. Usually, macrophages are the first line of defense against of the innate immune system to fight pathogens and to clearance foreign, aged, and damaged cells [2]. In chronic diseases, especially cancer and fibrosis, macrophages are usually polarized towards an immunosuppressive, alternative (M2-type) macrophage phenotype. It is mainly the cancer cells and the tumor associated stroma that promote macrophage repolarization towards the M2-type, i.e., tumor-associated macrophages (TAM). TAM dampen Th1 T cell or cytotoxic immune reactions, promote the rise of regulatory T-cells (Treg), or of other immune suppressive cells of the innate immune system, such as myeloid derived suppressor cells [2]. TAM are the source of various pro-angiogenic, pro-fibrotic, and T cell suppressive cytokines, e.g., vascular endothelial growth factor (VEGF) or transforming growth factor beta 1 (TGFβ1) [2].

Besides cancer, M2-type macrophages also play an important role in the development of organ fibrosis, which is considered the result of “wounds that do not heal” [3,4,5,6]. In the liver, fibrosis, and its end-stage cirrhosis, results from continuous inflammation, e.g., due to viral hepatitis, alcoholic or nonalcoholic steatohepatitis, or autoimmune liver diseases. Here, M2-type macrophages can subdue the chronic M1-type inflammation, while at the same time they promote scarring [7,8]. Cirrhosis gives rise to life-threatening complications linked to portal hypertension and liver failure. Importantly, cirrhosis is tightly linked to the development of liver cancer (hepatocellular carcinoma, HCC). Thus, the vast majority of HCC evolve in cirrhotic livers [9,10]. This association can be explained by the abnormal stroma of cirrhosis, favoring M2-type macrophages and the resulting immune suppression.

In liver fibrosis, M2 polarized macrophages mirror, therefore, many characteristics and qualities of TAM. Although the role of macrophages and their phenotype in liver fibrosis is complex, M2-type macrophages are pro-fibrotic in fibrosis progression and tumor stromal remodeling, and in cirrhotic livers, TAM by far outnumber other macrophage phenotypes and are correlated with poor prognosis in HCC [11,12,13].

Notably, even with a certain phenotype macrophages maintain their plasticity. Thus, M2-type macrophages can be re-educated to resume their original anti-cancer and anti-fibrotic M1-type polarization by small interfering RNA (siRNA) directed to macrophage receptors [14,15,16]. Such siRNA or DNA antisense strategies have also been effective in repolarizing the profibrotic M2 phenotype in mouse models of liver fibrosis [3,17,18,19].

Although RNA interference can be a powerful tool to target relevant transcripts in vivo, naked siRNA possesses unfavorable pharmacokinetics: After parenteral administration, siRNA is prone to rapid kidney clearance caused by its low molecular weight as well as immanent degradation by nucleases of the blood stream. Due to its highly negative charge, naked siRNA cannot penetrate cell membranes but triggers unspecific (innate) immune reactions [20]. Therefore, a suitable carrier system is required to overcome these limitations. Such system should also secure cell-specific delivery of siRNA, here to M2-type macrophages, to reduce the risk of off-target effects while achieving therapeutically relevant siRNA concentrations in the target cells.

To address these needs, we have established a block copolymer-based delivery system derived from reactive precursor polymers that self-assemble into micelles whose cores are then crosslinked and switched from hydrophobic to polycationic, allowing for efficient siRNA encapsulation [20,21,22]. Compared to other established siRNA delivery vehicles, these nanogels provide favorable characteristics for in vivo delivery, as demonstrated by us before [20,23,24,25,26]. Since M2 polarized macrophages overexpress the mannose receptor CD206, we have recently extended our non-targeted cationic nanohydrogel particle platform (NonNP) by applying heterotelechelic block copolymers with α-mannosyl end groups, resulting in α-mannose nanohydrogels (ManNP) [23]. In vitro, NonNP and ManNP showed no cytotoxicity and were preferentially taken up by the M2 polarized macrophages and demonstrated functional siRNA delivery by M2-specific knockdown of CSF-1 receptor (CSF-1R) mRNA, a relevant target in M2-type macrophages [14,23].

In this study, we applied ManNP and NonNP in vivo to mice with active liver fibrosis to assess their M2 macrophage-specific delivery. Dual near infrared dye labeling allowed in vivo monitoring of siRNA and NP delivery into organs and ex vivo immunohistochemical colocalization studies in the liver. We show that ManNP specifically targeted hepatic M2-type macrophages and demonstrated no organ or cellular toxicity, qualifying them as promising carriers for siRNA-directed macrophage repolarization in liver cancer and fibrosis.

## 2. Materials and Methods

### 2.1. Fabricationof α-Mannosyl Modified Cationic Nanohydrogel Particles

The fabrication of non-functionalized cationic nanohydrogel particles was performed as previously reported in detail [23]. In brief, the synthesis of ManNP was conducted equivalent to NonNPs and is described below for fluorescent labeled particles with Oregon Green 488. For in vivo biodistribution studies, (Non-)ManNPs were fabricated in analogy but using the near infrared dye NIR 800RS cadaverine, as fluorescent dye instead of Oregon Green 488.

All steps were conducted under argon atmosphere. In dry DMSO Man-P(MEO_3_MA)_18_-*b*-P(PFPMA)_30_ (40 mg; 3.24 µmol or 97.2 µmol reactive ester) as α-mannosyl end group modified amphiphilic block copolymer was dissolved (4 mL; 10 g/L) and treated with triethylamine (7.3 µL; 10% of total 0.54 mmol) and the corresponding fluorescent dye (89.8 µL of a 2.5 g/L stock solution in anhydrous DMSO; 0.45 µmol). The reaction mixture was vigorously stirred at room temperature for 16 h. Then, spermine (9.2 mg; 45.2 µmol) and once more triethylamine (68 µL; 90% of total 0.54 mmol) were added to initiate crosslinking. After stirring for additional 24 h at 50 °C, a sample was taken for ^19^F-NMR to determine complete conversion of the reactive ester units. To ensure further full conversion of all PFPMA units, spermine (18.3 mg; 90.5 µmol) was added once more, followed by 18 h stirring. Afterwards, the reaction mixture was dialyzed against Millipore water for several days, changing the water twice per day. The mixture was lyophilized and cationic nanohydrogel particles could be obtained as orange powder (25.9 mg; 2.63 µmol; 81%). For the detailed description of the synthesis and physiochemical analysis of the cationic nanohydrogel particles, refer to our previous work [23].

### 2.2. Loading of (Non-)ManNP with siRNA

SiRNA was complexed inside the NonNP at a weight ratio of 10:1 for 20 min at room temperature. SiRNAs were purified by high-performance liquid chromatography. A Cy5 labeled scrambled siRNA was used with the dye attached to the 5′ by a C6 amino spacer. It was purchased from bearing Dharmacon (Freiburg, Germany) with the following sequence:

5′-Cy5 GCAUCUGGCUUAAGGUGAAUU-3′

5′-PGUAUCUCUUCAUAGCCUUAUU-3′

### 2.3. Cell Culture: Cell Lines

The cell culture work has been performed as described by us in detail previously [23]. In brief, THP-1 monocytes, RAW macrophages or SVEC4-10 endothelial cells, and HepG2 human hepatocytes were cultured in RPMI Media 1640 (Sigma-Aldrich, Taufkirchen, Germany) or Dulbecco’s Modified Eagle’s Medium (DMEM) (Sigma-Aldrich, Taufkirchen, Germany) or Eagle’s Minimum Essential Medium (EMEM) (Sigma-Aldrich, Taufkirchen, Germany) supplemented with 10% fetal bovine serum (FBS), 1% penicillin/streptomycin, 1% l-glutamine, and 50 pM β-mercaptoethanol (Gibco; 31350–010) for THP-1 in 5% CO2 at 37 °C, respectively. The cells were passaged just before reaching confluency. For cell detachment, THP-1 and RAW cells were incubated in PBS buffer, without calcium and magnesium, supplemented with 10 mm EDTA, for 15 min on ice and then carefully detached by cell scraping, while Svec4-10 and HepG2 could be trypsinized with Trypsin-EDTA solution (Sigma-Aldrich, Taufkirchen, Germany). For differentiation into macrophages, THP-1 monocytes were incubated with 150 nM phorbol 12-myristate 13-acetate (Sigma-Aldrich, Taufkirchen, Germany) for 24 h followed by 12 h incubation in RPMI medium.

### 2.4. Cell Culture: Primary Macrophages—Bone Marrow Derived Macrophages (BMDM)

As described before in detail [23], bone marrow cells were extracted from the bone marrow of mice and matured in the presence of a macrophage colony-stimulating factor (M-CSF), a lineage-specific growth factor, into primary macrophages. Bone marrow was extracted from 6 to 9 weeks old Balb/c mice. In brief, mice were sacrificed by cervical dislocation, and femurs and tibias were isolated under sterile conditions. A 21G needle and a 10 mL syringe served to rinse out the marrow into cold PBS substituted with 2% heat inactivated FBS and 1% penicillin/streptomycin. Afterwards, cells were passed through a 70 µm cell strainer for cell separation and purification. Remaining red blood cells were lysed with red cell lysis buffer (eBioscience, San Diego, CA, USA). The resulting bone marrow cells were resuspended in Iscove’s Modified Dulbecco’s Medium (IMDM), 10% FBS, 1% penicillin/streptomycin, 1% L-glutamine containing 25 ng/mL monocyte colony-stimulating factor (M-CSF, ImmunoTools, Friesoythe, Germany). Approximately 5 million cells were plated out into bacterial culture plate in 10 mL (Sigmal-Aldrich/Merck, Darmstadt, Germany). On the following days, half of the media was replaced and substituted with fresh IMDM containing the same supplements. Cells were incubated for an additional 4 days in the presence of M-CSF for further differentiation into mature primary macrophages. M2- or M1-phenotype was obtained, when primary macrophages were incubated with fully supplemented IMDM containing 20 ng/mL IL-4 and IL-13 or 50 ng/mL interferon-γ and 0.1 µg mL^−1^ LPS (all from ImmunoTools, Friesoythe, Germany), respectively, for 24 h. Sub-culturing was performed as described above for THP-1 and RAW cells.

### 2.5. In Vitro Cytotoxicity

In vitro cytotoxicity of scrambled siRNA-loaded (non-)ManNP on THP-1 and SVEC4-10 was determined by MTT (3-(4,5-dimethylthiazol-2-yl)-2,5- diphenyltetrazolium-bromide) assay as described before [23]. In brief, cells were plated out into 96-well plates at a density of 6000–7000 cells per well and incubated in a fully supplemented medium. After 24 h, increasing concentrations of siRNA/NP (10:1 weight-to-weight ratio of (non-)ManNP to siRNA) corresponding to 5, 10, 20, 40, 60, 80, 100, 200, and 300 nm siRNA were added. After 48 h of incubation, 20 µL of MTT in PBS (4 mg/mL) was added to each well and incubated for 5 h. The medium was then carefully removed and 150 µL of DMSO was added. After full solubilization, optical density (OD) was measured on an Infinite M200Pro spectrofluorometer at 570 nm (TECAN, Männerdorf, Switzerland).

### 2.6. In Vitro Cellular Uptake of the siRNA-Loaded Carriers by Fluorescent Activated Cell Sorting Flow Cytometry (FACS)

Cells were plated out in 12-well culture plates at a density of 130,000 cells per well and incubated for 24 h. Cells were incubated with a final concentration of 25, 50, and 100 nM Cy5-labeled scrambled siRNA complexed with (Non-)ManNP (10:1 weight-to-weight ratio of (Non-)ManNP: siRNA) for 1 h at 37 °C. For normalization of potential autofluorescence of siRNA and nanoparticles, cells were incubated with a final concentration of 100 nM unlabeled scrambled siRNA complexed with unlabeled (Non-)ManNP (10:1 weight-to-weight ratio of (Non-)ManNP: siRNA) for 1 h in the cell incubator before FACS analysis. Control cells were incubated with equal volumes of PBS for 1 h at 37 °C. After 1 h incubation, culture medium was poured off, and the cells were repetitively washed with PBS. Detachment of the cells was performed as described above. Cells were then also stained with a viability dye (Fixable Viability Dye eFluor^®^ 506, eBioscience, San Diego, CA, USA) according to the manufacture’s protocol and stored at 4 °C under the exclusion of light. Finally, cells could be analyzed by flow cytometry (BD FACS Canto II, BD Bioscience, Canada, Mississauga) on the same day. 50,000 cells were measured per sample. The obtained data were analyzed with Flowing Software 2.5.0 (Perttu Terho, Turku Centre for Biotechnology, Finland).

### 2.7. Fibrosis Model

The conducted animal studies followed the approval of the local ethics committee on animal care (number 23 177-07/G 17-1-030, Government of Rhineland Palatinate, Germany). 8–10-week-old female Balb/c mice (body weight ~20–25 g) were bought from Charles River (Sulzfeld, Germany) and kept according the guidelines of the institute and the local government (12 h light-dark cycles at 25 °C and 40–60% humidity with humane care, access to regular chow and water ad libitum). Carbon tetrachloride (CCl_4_) (Sigma-Aldrich, St. Louis, US) diluted 1:1 in mineral oil (Sigma-Aldrich, St. Louis, US) was administered by oral gavage 3 times per week following an escalating dose protocol (first dose 0.875 mL/kg; 1.75 mL/kg week 1; 2.5 mL/kg week 2, 3, and 4), as reported in detail before [26]. Mice gavaged with pure mineral oil not containing CCl4 served as non-fibrotic healthy controls. At the described time points, mice were sacrificed by cervical dislocation.

### 2.8. In Vivo Imaging of Near Infrared (NIR) Labeled siRNA and Nanogel Particles

At week 4 of CCl_4_ treatment, mice (*n* = 5 per group) were anesthetized with isoflurane gas and treated via tail vain injection with 2 mg/kg Cy5-labeled scrambled siRNA loaded into NIR dye RS800 labelled (Non-)ManNP (10:1 weight-to-weight ratio of (Non-)ManNP: siRNA) for in vivo NIR fluorescence imaging by an IVIS (in vivo imaging spectrum) system (Caliper Life Sciences, Hopkinton, US). At the given time points, mice were anesthetized with isoflurane gas and transferred into the imaging chamber according the manufactures protocol. Picture integration time of the fluorescence source was set to 4 s. Recording parameters were set for excitation at 745 nm and emission at 800 nm to visualize IRDye RS800 (near infrared dye) labeled nanohydrogel particles, while for detecing Cy5-dye-labeled scsiRNA they were adjusted to excitation at 640 nm and emission at 700 nm.

### 2.9. Ex Vivo Imaging of Organs

24 h after Cy5-scsiRNA-loaded (Non-)ManNP injection, mice were sacrificed, and organs were harvested and put into the imaging chamber. Image setup was adjusted in analogy to the settings described above. Ex vivo signal quantification of Cy5-scsiRNA and NIR-NonNP in the isolated organs could then be quantified by using Live Image software from PerkinElmer. Organs were carefully circumscribed by region-of-interests (ROIs), and then their fluorescence was quantified as the average of area-normalized radiant efficiency.

### 2.10. Production of Liver Single Cell Suspensions

After imaging, livers were processed according to obtain single cell suspensions following the manufactures’ protocol (Milteny, Bergisch Gladbach, Germany) as described by us previously [25,26]. Briefly, livers were washed with PBS and, after extraction of the gall bladder, digested with 5000 U/mL of collagenase IV (C5138, Sigma-Aldrich) in Krebs-Ringer-Buffer, pH 7.4. The livers were carefully minced with a gentleMACS™ dissociator (Milteny, Germany, Bergisch Gladbach) and incubated for 30 min at 37 °C. This step was repeated once more before the cell suspension could be filtered over a 100 µm cell strainer to remove undigested tissue clumps. The filtered cell suspension was afterwards centrifuged at 300× *g* for 10 min at 4 °C, and thus, parenchymal and non-parenchymal cells could be collected. The supernatant was poured off, and the remaining cell pellet was then resuspended in 5 mL 1× Red Blood Cell Lysis Solution (Milteny, Germany, Bergisch Gladbach) for 5 min at RT to remove the remaining erythrocytes. Finally, remaining live cells were washed 3 times with PBS and further processed for FACS analysis.

### 2.11. In Vivo Cellular Uptake of Cy5-siRNA-Loaded NIR-Labelled NonNP

To quantify the in vivo cellular uptake of Cy5-labeled scsiRNA complexed with NIR-labeled NonNP via FACS analysis, isolated liver cells obtained from mice injected with 2mg/kg scsiRNA/NonNP were stained with fluorescent-labeled antibodies. The following antibodies were applied according the manufacture’s protocol for intra/extracellular staining: macrophages: F4/80-PE (clone BM8) or CD11b-BV421 (clone M1/70, both from eBiolegend, San Diego, CA, USA); hepatocytes: albumin-PE (Clone #188835, R&D, Boston, MA, USA) and endothelial cells; CD31-PE (MEC13.3, eBiolegend, San Diego, CA, USA); granulocytes: Gr1+ (RB6-8C5, MEC13.3, eBiolegend, San Diego, CA, USA); NKT-cells: CD4-FITC (GK1.5) and NK1.1 (PK136, both from eBiolegend, San Diego, USA); dendritic cells: F4/80-PE (clone BM8) and CD11c-BV421 (clone N418, both from eBiolegend, San Diego, CA, USA); macrophages: M2 phenotype CD45-FITC (clone 30F11) combined with F4/80-PE (clone BM8) and CD206-BV421 (C068C2, all from eBiolegend, San Diego, CA, USA). Moreover, eFluor 506 (eBioscience, San Diego, CA, USA) was used for live/dead staining. Afterwards, cells were fixed with 4% formaldehyde/PBS for 15 min at 37 °C and analyzed by a BD FACS Canto II (BD Bioscience, Canada, Mississauga) Flow cytometer. 10,000–50,000 cells were measured per staining. The obtained data were analyzed by Flowing Software 2.5.0 (Perttu Terho, Turku Centre for Biotechnology, Finland).

### 2.12. Confocal Laser Scanning Microscopy of CD206 Immunofluorescent Stained Liver Cryosections from Mice Treated with Cy5-siRNA-Loaded NIR-(Non-)ManNP

Directly after sacrifice, parts of the middle and right liver lobe from mice treated with 2 mg/kg scsiRNA/(Non-)ManNP were embedded in OCT compound from Sakura (Staufen, Germany) and stored at −20 °C. Liver cryosections were cut at 6 µm with a Leica CM 1950 cryostat (Wetzlar, Germany) and mounted on superfrost ultra plus adhesion histological slides from Thermo Fisher Scientific (Schwerte, Germany). Slides were stored at −70 °C for subsequent staining. Cryo slides were rehydrated with PBS at room temperature for 20 min, fixed with fixation buffer (intracellular fixation&permeabilization buffer set, eBioscience, Frankfurt, Germany) for 10 min and washed three times with a permeabilization buffer. Tissues were surrounded with a hydrophobic barrier using a Dako pen from Agilent (Santa Clara, CA, USA). Afterwards, sections were blocked with 5% donkey serum in a permeabilization buffer for 1 h at room temperature and incubated overnight at 4 °C with CD206-FITC antibodies (C068C2, eBioscience, San Diego, CA, USA) diluted 1:100 in permeabilization buffer. After 12 h, slides were washed with a permeabilization buffer and H_2_O, covered with coverslips after prior application of antifade mounting medium, and stained with DAPI from VECTASHIELD (Orton Southgate, UK).

## 3. Results and Discussion

### 3.1. α-Mannosyl-Modified Cationic Nanohydrogel Particles

**Figure 1:**
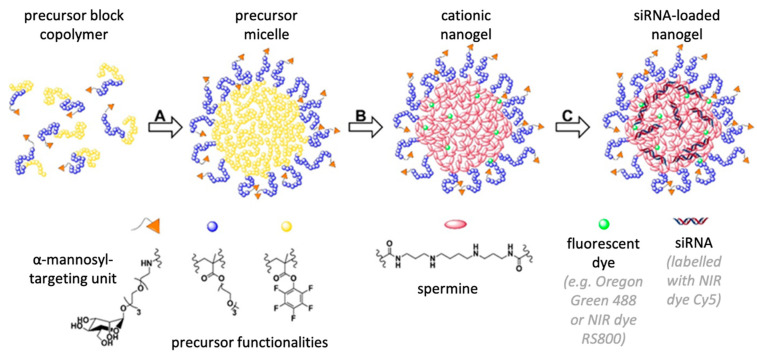
Preparation scheme of α-mannosyl-functionalized cationic nanohydrogels (ManNP): RAFT polymerization with an α-mannosyl-functionalized chain transfer agent yielded amphiphilic reactive ester block copolymers. (**A**) In aprotic solvents, co-block polymers spontaneously assemble into micellar structures with reactive ester cores. (**B**) Cross-linking of these precursor micelles is obtained by spermine, a bifunctional oligoamine, yielding ManNP with a cationic core. Fluorescent labeling could be achieved when adding dyes functionalized with a primary amine. (**C**) Loading of siRNA into carrier for targeted delivery is obtained by electrostatic interactions of the negatively charged siRNA and the positively charged nanohydrogel cores.

α-mannosyl-functionalized cationic nanohydrogel particles (ManNP) were prepared as described earlier (Figure 1) [23]. In brief, α-chain end mannose-functionalized amphiphilic block copolymers were obtained by controlled RAFT polymerization, using an α-mannosyl-functionalized chain transfer agent (Man CTA). The amphiphilic diblock copolymer of a hydrophilic PEG-like and a hydrophobic reactive ester block from polymerized triethylene glycol methyl ether methacrylate (MEO_3_MA) or pentafluorophenyl methacrylate (PFPMA), respectively, was dissolved in polar aprotic solvents (e.g., DMSO) and spontaneously self-assembled into micelles. The inner core of the micelles was stabilized by cross-linking with spermine, a bifunctional oligoamine, and labeled with fluorescent dyes, containing a primary amine as functional group. After cross-linking, the stable particles efficiently complex anionic siRNA due to electrostatic interactions. For efficient targeting of the mannose receptor CD206, the mannose residue of ManNP was located on the particle’s surface, following the controlled radical polymerization process. This feature was already demonstrated before by nanogel binding to Concanavalin A (Con A) [23].

### 3.2. In Vitro Evaluation of siRNA-Loaded NonNP and ManNP in Human Macrophages

Prior to in vivo evaluation, Non(Man)NP loaded with scrambled Cy5-siRNA (non-specific siRNA) (weight-to-weight (*w/w*) ratio of NP to siRNA 10:1—at this ratio all siRNA is complexed and affords charge-neutralized carriers [23], compare Table 1—were tested for in vitro cytotoxicity in human macrophage differentiated THP-1 monocytes and murine endothelial cells (SVEC4-10), reflecting characteristics of liver resident macrophages and liver sinusoidal endothelial cells (LSECs), respectively. Both non-parenchymal cell types play important roles in liver fibrogenesis and cancer [10].

**Figure 2:**
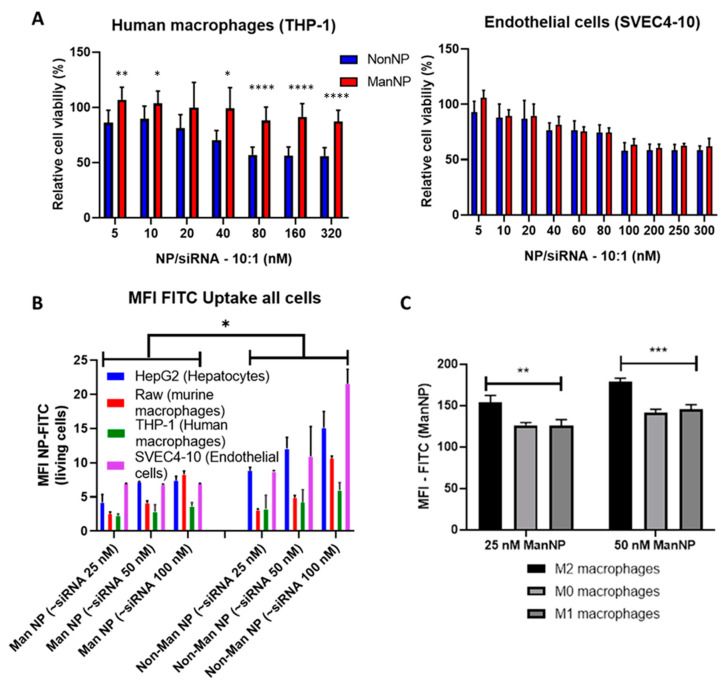
In vitro performance of ManNP and NonNP. (**A**) Cell viability of THP-1 macrophages and SVEC4-10 liver endothelial cells exposed to escalating doses of scrambled siRNA (scsiRNA)-loaded ManNP/NonNP showing that both scsiRNA loaded carriers were well tolerated by the cell-lines, as determined by the MTT assay (10:1 weight-to-weight ratio *w/w* of (Non-)ManNP: siRNA, incubation time 72 h, *n* = 2, *, **, **** *p* < 0.05, 0.001, 0.00001—NonNP vs. ManNP). (**B**) Cellular uptake of Cy5-scsiRNA-loaded and Oregon Green 488 labeled (Non-)ManNP (488-(Non-)ManNP) in human hepatocytes (HepG2), murine and human macrophages (RAW and THP-1 macrophages), and liver endothelial cells (SVEC4-10) at 25, 50, and 100 nM siRNA concentrations after 1 h incubation, followed by flow cytometry assessment (exemplary dot plots shown in Figure S2, *n* = 3, * *p* < 0.05) (**C**). Cellular uptake of scsiRNA-loaded 488-ManNP in native M0 and M2-, M1-polarized primary macrophages at 25 and 50 nM siRNA concentrations after 1 h incubation and analyzed by flow cytometry. Cellular uptake of 488-ManNP was significantly higher in M2 polarized macrophages compared to M0- and M1-polarized macrophages (MFI: mean fluorescence intensity of 488-(Non-)ManNP loaded with (Cy5-)scsiRNA, *n* = 3, ** *p* < 0.001, *** *p* < 0.0001).

As determined by the MTT assay to verify cell viability, both carriers ManNP and NonNP were tolerated up to high siRNA concentrations (>100 nM siRNA) in THP-1 and SVEC4-10 cells. Interestingly, in THP-1 cells ManNP showed less tendency for cytoreductive effect especially at higher siRNA concentrations (>100 nM siRNA) compared to NonNP (Figure 2A).

Next, Oregon Green 488 fluorescently labeled particles loaded with NIR Cy5-labelled siRNA exhibited a dose-dependent uptake in cells (Figure 2B). Here, NonNP were dose-dependently taken up by endothelial cells (SVEC4-10), followed by hepatocytes (HepG2) and macrophages, while uptake was lower in human THP-1 vs. murine RAW macrophages. When comparing uptake efficiency of ManNP vs. NonNP, the non-functionalized particles exhibited a significantly higher uptake (*p* < 0.05) than ManNP, suggesting that functionalization with mannose may stabilize the nanocarriers and prevent them from non-specific cellular uptake. But, cellular uptake was higher for ManNP in M2 polarized macrophages that overexpress the mannose receptor CD206, compared to native or M1 polarized macrophages, as demonstrated by us in our earlier work (Figure 2C) [23]. Altogether, these findings suggest that mannose functionalization is a valuable tool to shield the carriers and prevent them from unspecific uptake in non-targeted macrophage phenotypes and other (non-)parenchymal cells, while maintaining their good biocompatibility.

Based on these promising in vitro results for M2 phenotype specific targeting, the novel siRNA carriers qualified for further biological evaluation in vivo.

### 3.3. In Vivo Biodistribution on the Organ Level

The in vivo biodistribution of RS800-NonNP loaded with Cy5-scsiRNA was tested in a chemically induced liver fibrosis mouse model, which leads to an increase in M2-type macrophages. Non-functionalized nanogels had already been studied in a similar liver fibrosis model earlier before, where the siRNA-loaded carriers showed effective accumulation in liver and fibrosis relevant to nonparenchymal cells [25,26]. In the current study, we aimed to study whether we could maintain liver targeting but guide the mannosylated carriers more into immunosuppressive and profibrotic M2-type macrophages, which represent a key target for future RNA interference-based antifibrotic and anti-cancer immunotherapies.

**Figure 3:**
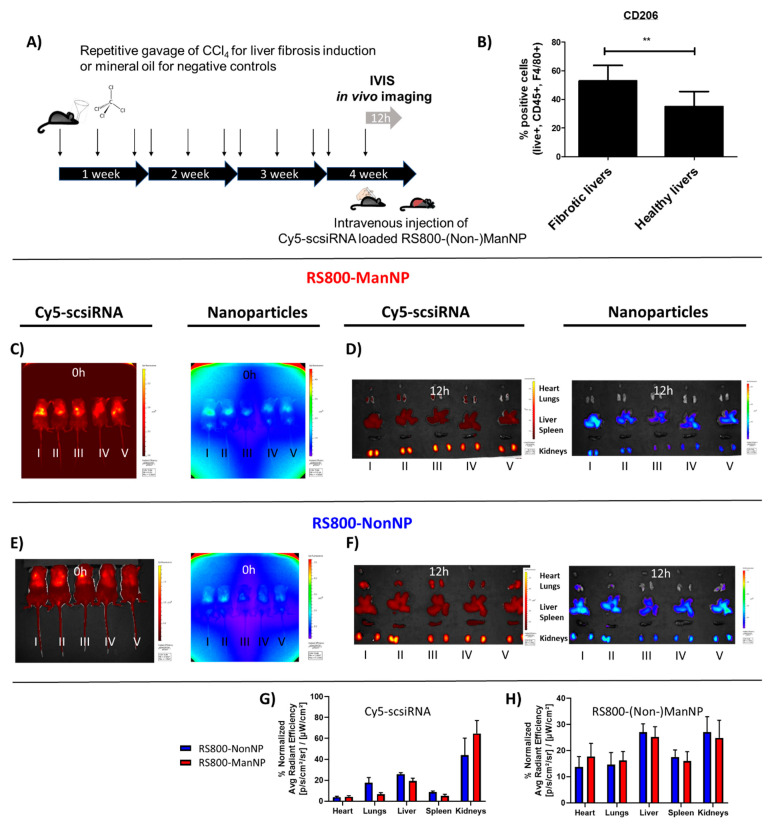
In vivo biodistribution of Cy5 labeled scsiRNA-loaded NIR-labeled RS800-(Non-)ManNP in liver fibrotic mice on the organ level. (**A**) CCl_4_ liver fibrosis mouse model: BALB/c mice were gavaged three times weekly with escalating doses of CCl_4_ over a period of 3.5 weeks. (**B**) FACS analysis of a liver single cell suspension obtained from healthy and liver fibrotic mice. Profibrotic CD206+ macrophages are significantly higher in fibrotic compared to healthy livers (*n* = 5 per group, ** *p* < 0.001). (**C**,**E**) NIR in vivo imaging of RS800-ManNP/NonNP loaded with Cy5-scsiRNA directly after injection (*t* = 0 h): Carrier and siRNA accumulated primarily in the fibrotic livers. (**D**,**F**) Corresponding ex vivo imaging of Cy5-scsiRNA-loaded RS800-NonNP in organs harvested 12 h after IV injection (organs of mice treated with PBS alone served as autofluorescence controls): both particles and their cargo accumulated primarily in the livers (Figure S1). (**G**,**H**) Quantification of ex vivo organ fluorescence as shown in extracted organs above. Both siRNA cargo and particles efficiently accumulated in the liver, while biodegraded particle fragments and siRNA were excreted via the kidneys.

Mice were gavaged with escalating doses of carbon tetrachloride (CCl_4_) three times per week over three and a half weeks, resulting in a 2-fold increase in hepatic collagen deposition compared to control mice that received the vehicle (mineral oil) alone, as reported by us earlier (Figure 3A) [25,26]. This model allowed us to generate fibrotic livers with a significantly increased number of CD206^+^ macrophages compared to the nonfibrotic control livers and study the effect of α-mannosyl-derived targeting (Figure 3B).

As reported before, NonNP are well-defined siRNA carriers that exhibit no aggregation in blood and show a preferential accumulation in fibrotic liver after intravenous application [21,22]. Considering the proven good biocompatibility of NonNP in vitro and in vivo, application of the ManNP carriers was justified to study siRNA targeting efficiency to M2-macrophages in this fibrosis model.

12 h before sacrifice, fibrotic mice received a single intravenous injection of 2 mg/kg Cy5-scsiRNA loaded near infrared (NIR) dye RS800-labeled ManNP or NonNP. Biodistribution of the double NIR dye-labeled complexes was monitored in vivo at 0, 2, and 12 h after injection using fluorescent in vivo NIR imaging (Figure S1A,B). In analogy to RS800-NonNP, RS800-ManNP together with their Cy5-scsiRNA cargo were rapidly sequestrated in the fibrotic livers of the mice, while non-complexed free Cy5-scsiRNA was instantly eliminated via the kidneys, which is in agreement to earlier studies (Figure 3C,E) [26].

12 h after the injection, mice were sacrificed, and their organs prepared for ex vivo imaging, providing a better visualization of the distribution of the siRNA and the NP in the individual organs. In line with the in vivo imaging results, both particle species mediated an efficient delivery of siRNA to the fibrotic liver of the mice, while other organs, including lungs and spleen, that often show a high accumulation of other nanoparticles were largely spared (Figure 3D,F). After biodegradation of particles and likely siRNA breakdown, products were excreted mainly via the kidneys, causing a strong ex vivo fluorescence signal in kidneys, as observed before (Figure 3G,H) [25,26]. In vivo and ex vivo biodistribution of siRNA-loaded carriers were also assessed in healthy mice. Equal to fibrotic mice, both particles were efficiently sequestered in healthy livers, suggesting that Cy5-scsiRNA delivery via RS800-ManNP is also feasible in non-fibrotic livers. (Figure S1C,D).

### 3.4. In Vivo Biodistribution on the Cellular Level

**Figure 4:**
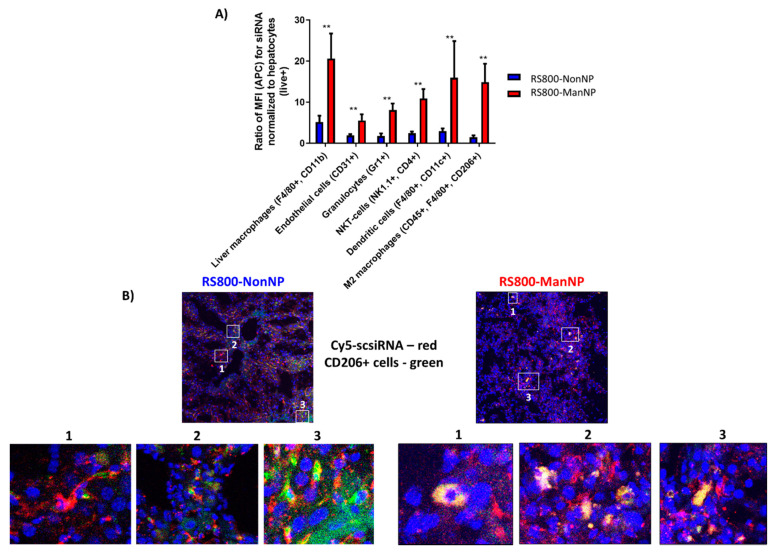
In vivo uptake of Cy5-scsiRNA-loaded RS800-(Non-)ManNP on cellular level. Fibrotic livers were harvested 12 h after a single IV injection of Cy5-scsiRNA-loaded RS800-(Non-)ManNP (2 mg/kg siRNA). (**A**) In vivo cellular uptake of both carriers (RS800-NonNP and -ManNP) in (non-)parenchymal liver cells as assessed by FACS analysis of single cell suspensions obtained from harvested livers. Cell-specific uptake in relation to hepatocytes as most abundant liver cells was significantly enhanced for RS800-ManNP in M2 polarized macrophages (CD45^+^, F4/80^+^ and CD206^+^) after normalization to hepatocyte uptake (** *p* < 0.001). (**B**) Confocal fluorescence laser microscopy of liver cryosections obtained from fibrotic mice. Liver sections were stained for the mannose receptor CD206 (green) and Cy5-siRNA (red). Colocalization of carriers with CD206+ cells is indicated by yellowish staining. Sections of fibrotic mice injected with PBS as vehicle control served to normalize for background fluorescence (Figure S4).

As almost similar amounts of particle and payload could be found in fibrotic livers of the CCl_4_-treated mice for both RS800-NonNP and RS800-ManNP, we next studied for their in vivo cell-specific uptake using flow cytometry of liver-derived single-cell suspensions obtained from the fibrotic mice. In view of the nontargeted Cy5-scsiRNA/RS800-NonNP particles being generally taken up by a spectrum of different cell types (Figure S3), we compared cellular uptake in relation to uptake into hepatocytes that represent the abundant liver cells (Figure 4A). Here, RS800-ManNP were preferentially taken up by CD206 expressing M2-type macrophages, but also other relevant immune cells that express other mannose-recognizing scavenger receptors (e.g., DC-SIGN, L-SIGN, Endo180, Langerin, or mannose binding lectins) that are mainly found on antigen-presenting immune cells, and that usually contribute to the immune suppressive microenvironment [27]. Taken together, as indicated in vitro, α-mannosyl functionalization appears to protect from unspecific uptake in non-targeted cell populations and lead to increased uptake in the desired cell types also in vivo (Figure S3).

Co-localization of the nanocarriers with CD206+ expressing immune cells including M2-type macrophages was also assessed by confocal laser microscopy of liver cryosections stained for CD206. Here, Cy5-scsiRNA/RS800-ManNP exclusively colocalized with CD206+ macrophages, illustrated by yellowish staining when CD206+ cells (green), were superimposed with Cy5-scsiRNA (red) (Figure 4B). In contrast, colocalization of RS800-NonNP carrying Cy5-scsiRNA with CD206^+^ M2-type macrophages was lower, resulting in only occasional yellowish co-staining in the liver cryosections (Figure 4B). Control cryosections of buffer-treated mice showed neither a fluorescent signal for Cy5-scsiRNA nor for CS800-(Non-)ManNP (Figure S4).

Thus, ManNP promote efficient uptake into mannose-receptor expressing immune cells in fibrotic livers, including M2 macrophages (CD206^+^). Additionally, the introduction of this carbohydrate functionalization further prevented uptake in non-targeted (non-)parenchymal liver cells. Therefore, ManNP can be considered to promote selective siRNA delivery to M2-type macrophages (and some other mannose receptor expressing immune cells) in vivo.

### 3.5. In Vivo Biocompatibility

Finally, high biocompatibility and lack of overt organ toxicity of the carriers in vivo was assessed by analysis of serum markers for liver inflammation (aspartate transaminase, alanine transaminase), cholestasis (alkaline phosphotase, gamma-glutamyltransferase, total bilirubin), and kidney function (creatinine) in fibrotic and healthy mice (Figure S5). These safety parameters did not change 12 h after injection of siRNA-loaded (Non-)ManNP at therapeutic concentrations (2 mg/kg). The lack of acute toxicity of ManNP confirms that the introduced surface modification with mannose did not compromise the good biocompatibility of the non-derivatized nanohydrogel particles and qualifies them for future RNA interference-based antifibrotic and anti-cancer immune therapies. Ongoing studies will evaluate various siRNA targets to induce M1-type macrophage polarization and their outcome on liver cancer and fibrosis progression.

## 4. Conclusions

In this study α-mannosylated siRNA nanohydrogel carriers were evaluated for specific targeting of profibrotic M2 polarized macrophages through the overexpressed mannose receptor CD206 in liver fibrotic mice. Carriers were synthesized and characterized as described by us in detail previously, yielding chemically well-defined nanohydrogel particles that carry a high degree of α-mannosyl functionalization on their surface [23].

To qualify for in vivo application, siRNA carriers had to meet three primary requirements of in vitro screening. First, cytotoxic or pro-inflammatory effects of particles had to be ruled out at concentrations relevant for in vivo therapy. Here, ManNP were tested in the human macrophage cell line THP-1 that mirrors characteristics of liver resident macrophages. Equal to murine cells, α-mannosyl functionalization did not compromise THP-1 cell viability at reasonable concentrations (25–50 nM siRNA). ManNP were even better tolerated by THP-1 cells up to very high siRNA concentrations (>320 nM) than their non-functionalized counterparts. Secondly, carriers had to demonstrate preferred uptake in disease relevant cells. ManNP showed a dose-dependent uptake by cells relevant for fibrogenesis. However, in comparison to NonNP, unspecific cellular uptake in liver epithelial cells and fibroblasts was lower for mannose functionalized carriers, which may be due to the mannose corona that prevents unspecific uptake in non-targeted cells. This will be an advantage over non-functionalized NPs, reducing, e.g., unwanted off-target side effects. Thus, relative uptake of ManNP was higher in M2 polarized macrophages, overexpressing the mannose receptor CD206, than in other cell types, as demonstrated by us before [23]. Third, knockdown efficiency of the siRNA-loaded carriers needs to be robust for the target transcript in relevant cells. Here, anti-*CSFR1* siRNA-loaded ManNP have already been demonstrated to induce solid knockdown in M2 polarized primary macrophages, while the (low CD206 expressing) M1 phenotype was not affected [23]. Taken together, our ManNP met all these key criteria for their application in vivo.

In the current in vivo liver fibrosis model enriched in M2 macrophages, fluorescently labeled ManNP loaded with Cy5-scsiRNA were rapidly sequestrated in the fibrotic liver after intravenous administration comparable to their non-functionalized analogue. In vivo biodistribution of the carriers was independent from fibrosis as accumulation of the complexes was also observed in healthy mice. Since the carriers also accumulate in livers of healthy mice, in vivo siRNA delivery to M2 macrophages appears feasible even at the early stages of chronic liver disease, allowing preventive treatment.

In these in vivo studies, we also looked at the cellular uptake of both ManNP and NonNP in those fibrotic livers. As predicted by the previous in vitro studies, cellular uptake of ManNP in M2-type macrophages was higher than in other nonparenchymal, i.e., fibroblastic and endothelial liver cells, and was also increased compared to their non-functionalized counterparts, while particles without mannose seem to be randomly internalized independent from the (macrophage) phenotype.

In summary, the introduced mannose surface functionalization prevented both unspecific uptake in non-target cells and significantly enhanced uptake in M2 polarized macrophages with a high expression of CD206 in vivo. Moreover, the introduced functionalization did not compromise the good biocompatibility of the nanohydrogel particles. Finally, in view of effective siRNA delivery by the non-functionalized NP in vivo [25,26], our ManNP qualify as improved nanocarriers for M2 macrophage-specific siRNA delivery. As the next generation of nanohydrogel particles, ManNP represent a promising siRNA carrier platform for further translational therapeutic studies for repolarization of immunosuppressive and profibrotic M2-type macrophages.

## Figures and Tables

**Table 1:** Characterization data of α-mannosylated and non-mannosylated cationic nanohydrogel particles [23].

Sample	D_h_ [nm] (and PDI) ^1^	ζ-Potential [mV] (NP Only) ^2^	ζ-Potential [mV] (NP:siRNA = 10:1) ^3^
ManNP	19.0 (0.19)	38.4 ± 1.8	1.7 ± 0.1
NonNP	23.8 (0.13)	38.2 ± 0.6	−1.1 ± 0.2

^1^ Hydrodynamic diameter and polydispersity determined by multi-angle DLS; ^2^ zeta potential of empty cationic nanohydrogels determined in H_2_O; ^3^ zeta potential of siRNA-loaded nanogels at 10:1 (*w/w*) of NP to siRNA. The summarized data have already been reported previously [23].

## Supplementary Materials

The following are available online at https://www.mdpi.com/2073-4409/9/8/1905/s1, Figure S1: IVIS in vivo and ex vivo imaging of NIR-Cy5-scsiRNA loaded NIR-labeled RS800-(Non-)ManNP, Figure S2: Gating strategy for vitro cellular uptake determined by flow cytometry analysis, Figure S3: In vivo cellular uptake of RS800-NonNP and RS800-ManNP in (non-)parenchymal liver cells, Figure S4: Confocal laser microscopy of a liver cryosection from PBS treated control mice. Figure S5: Serum markers of CCl_4_ treated fibrotic and non-treated healthy mice after injection with Cy5-scsiRNA loaded RS800-(Non-)ManNP (2 mg/kg siRNA).
