# Supplementary material for: In Vivo siRNA Delivery to Immunosuppressive Liver Macrophages by α-Mannosyl-Functionalized Cationic Nanohydrogel Particles
Source: Cells. 2020 Aug 15;9(8):1905. doi: 10.3390/cells9081905 (PMC7465192; doi:10.3390/cells9081905)
Supplement: Supplementary file 1 [file cells-09-01905-s001.pdf]

In vivo siRNA Delivery to Immunosuppressive Liver Macrophages by α-Mannosyl-Functionalized Cationic Nanohydrogel Particles

Leonard Kaps, Nadine Leber, Adrian Klefenz, Niklas Choteschovsky, Rudolf Zentel, Lutz Nuhn, Detlef Schuppan**

Supplementary Information

Dr. L.Kaps‡
‡ *The data of the NPs' biological evaluation is part of Leonard Kaps' medical doctoral thesis*
First Department of Medicine, University Medical Centre of the Johannes Gutenberg-University, Mainz, Germany, University Medical Centre of the Johannes Gutenberg-University, Langenbeckstrasse 1,
55131 Mainz, Germany

Dr. N. Leber, Prof. Dr. R. Zentel
Department of Chemistry, Johannes Gutenberg-University of Mainz, Duesbergweg 10-14, 55128 Mainz, Germany

Dr. L. Kaps‡, Adrian Klefenz, Niklas Choteschovsky, Prof. Dr. Dr. D. Schuppan*
Institute of Translational Immunology and Research Center for Immunotherapy (FZI), University Medical Center of the Johannes Gutenberg-University Mainz, Obere Zahlbacher Str. 63, 55131 Mainz, Germany
E-mail: detlef.schuppan@unimedizin-mainz.de

Dr. L. Nuhn*
Max-Planck-Institute for Polymer Research, Ackermannweg 10, 55128 Mainz, Germany
E-mail: lutz.nuhn@mpip-mainz.mpg.de

Prof. Dr. Dr. D. Schuppan
Division of Gastroenterology, Beth Israel Deaconess Medical Center, Harvard Medical School, 330 Brookline Avenue, Boston, MA 02215, USA

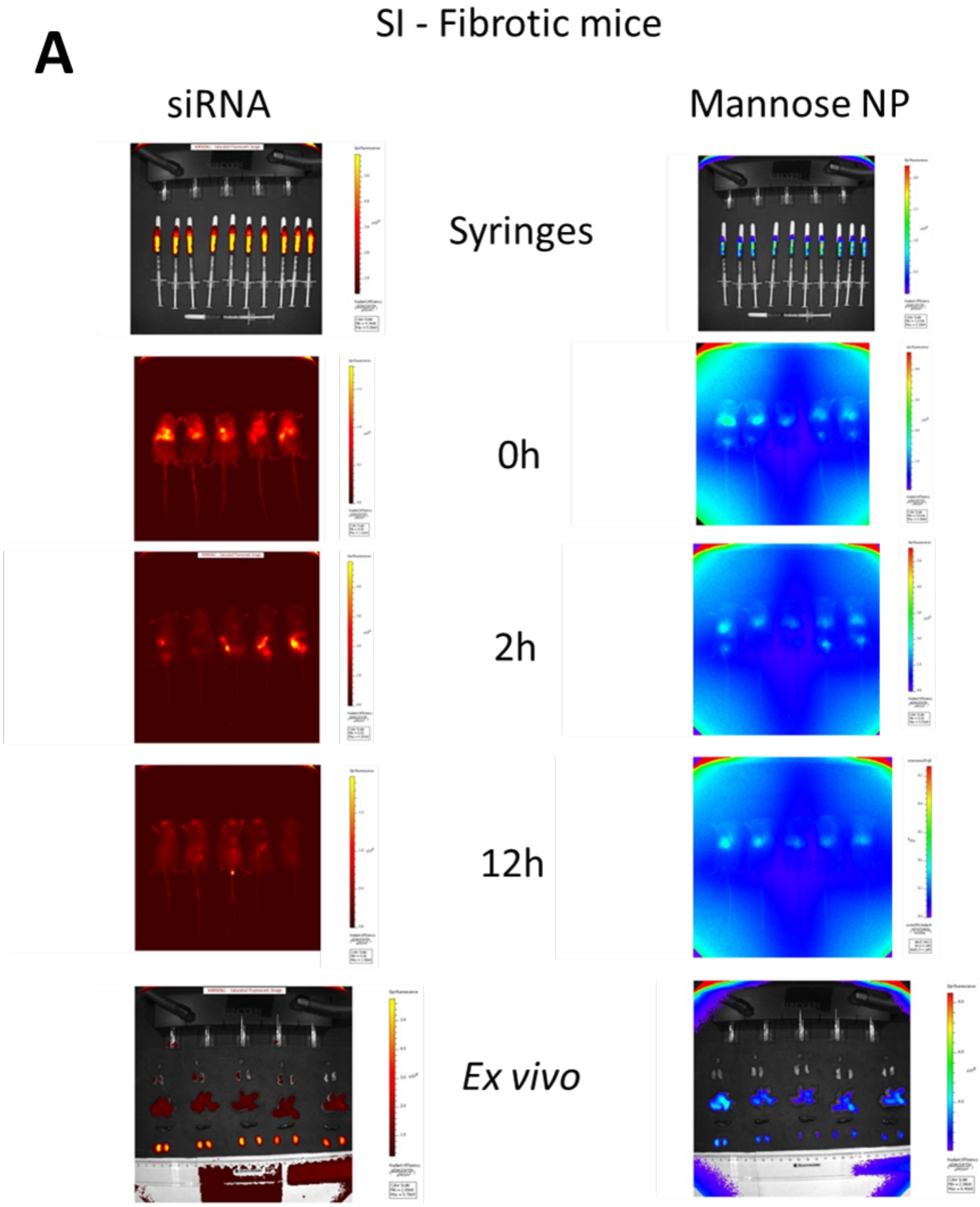
A
SI - Fibrotic mice
siRNA
Mannose NP
Syringes
0h
2h
12h
Ex vivo

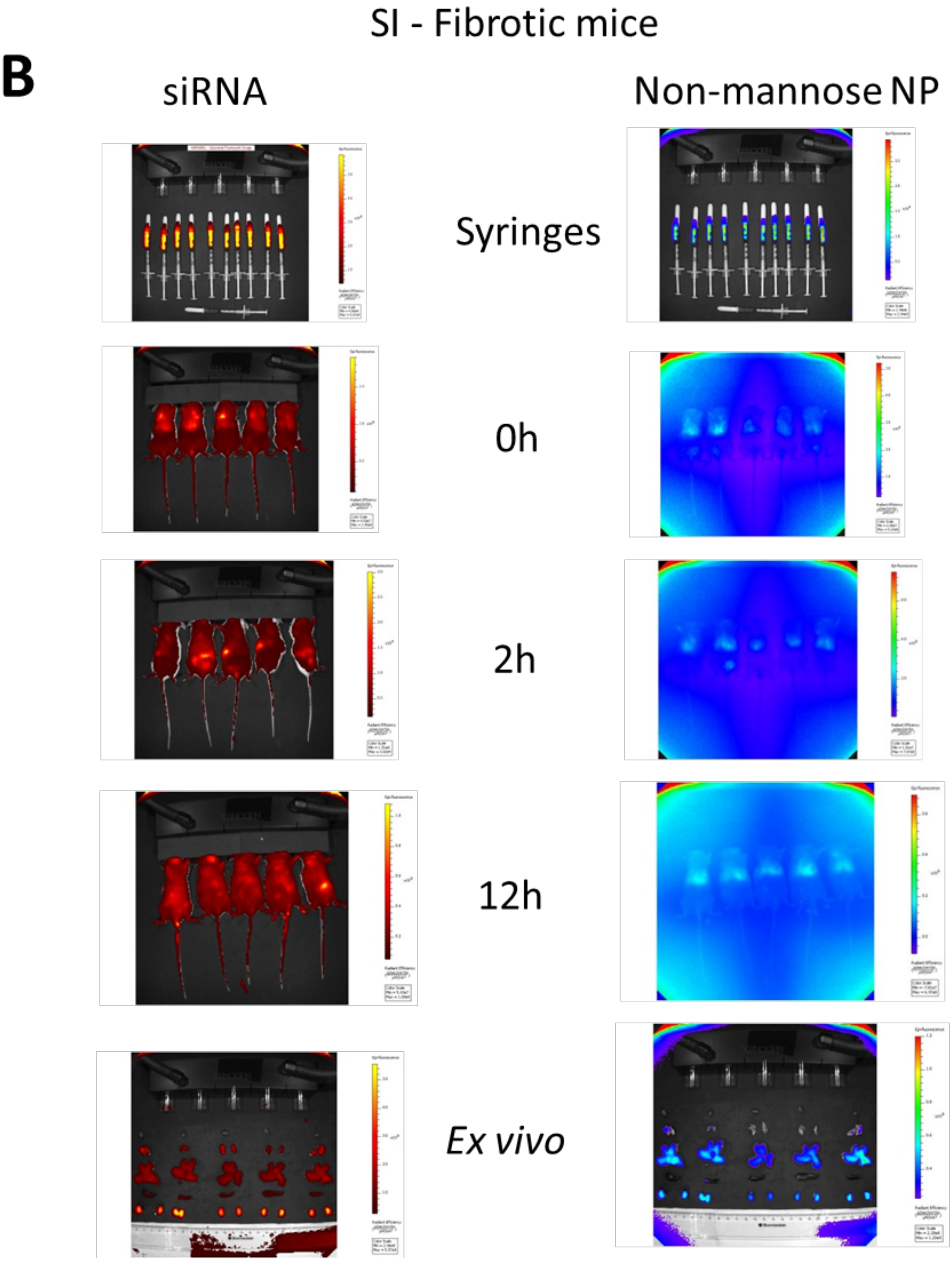
SI - Fibrotic mice
B
siRNA
Non-mannose NP
Syringes
0h
2h
12h
Ex vivo

C

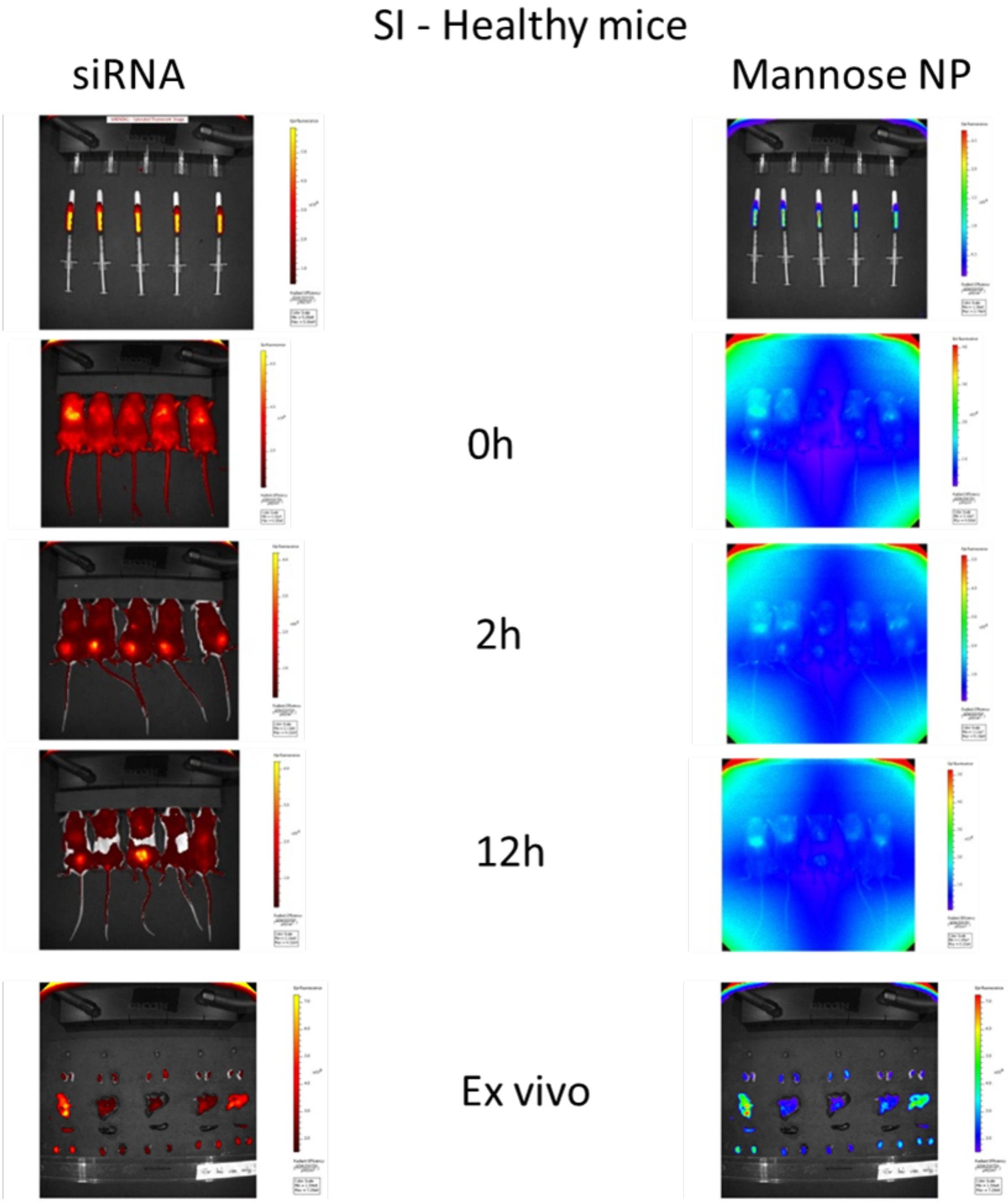

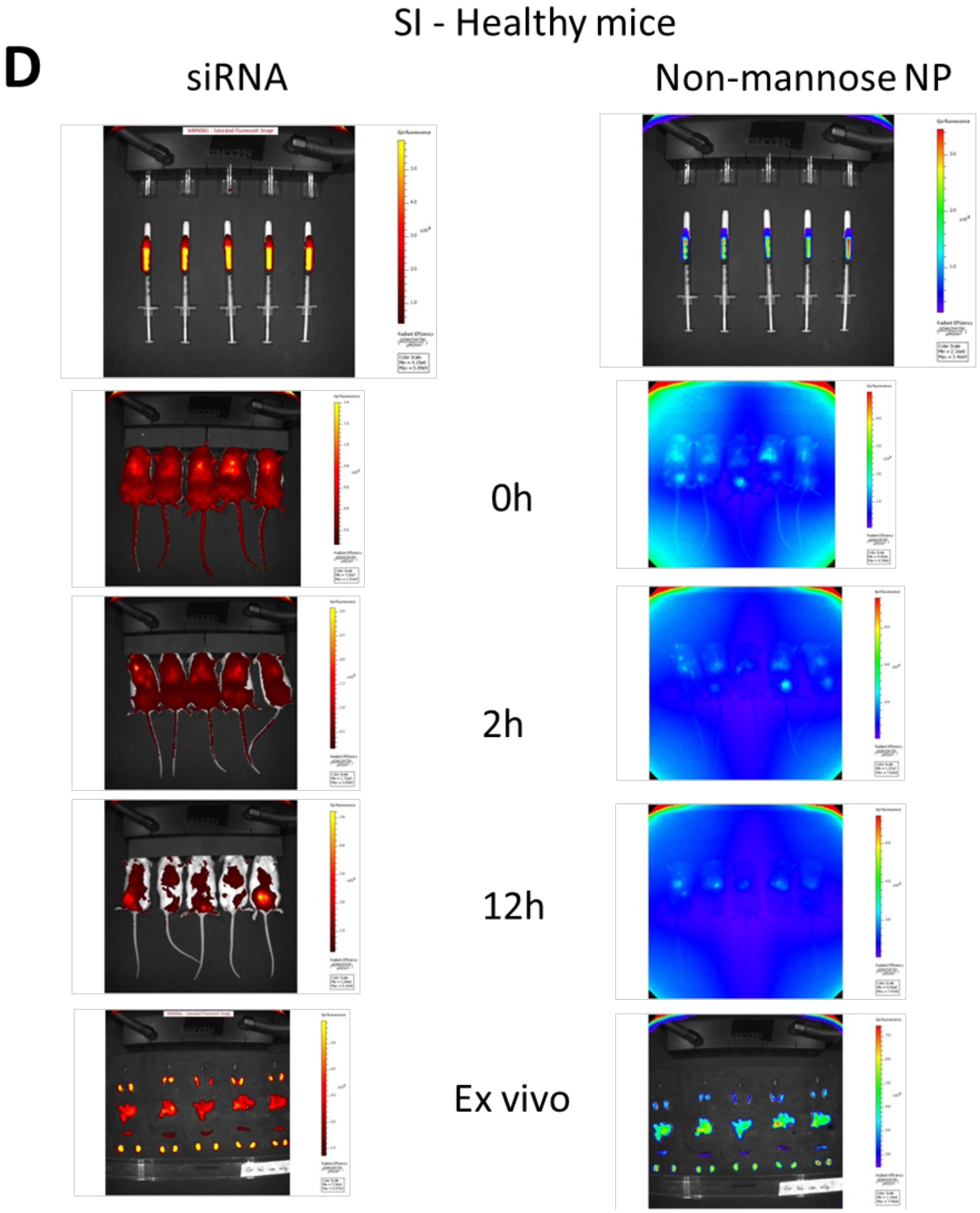

Figure S1. IVIS in vivo imaging of NIR-Cy5-scsiRNA loaded NIR-labeled RS800-(Non-)ManNP at 0, 2 and 12 h after i.v. injection in healthy and liver fibrotic mice (syringes with complexes shown). Ex vivo imaging of the corresponding organs (order: heart, lungs, liver, spleen and kidneys) after 12 h of injection. After i.v. injection, both siRNA cargo and NonNP primarily colocalized in the liver, while breakdown products of carrier and siRNA cargo were mainly excreted via the urinary tract, resulting in a strong fluorescent signal also in the kidneys.

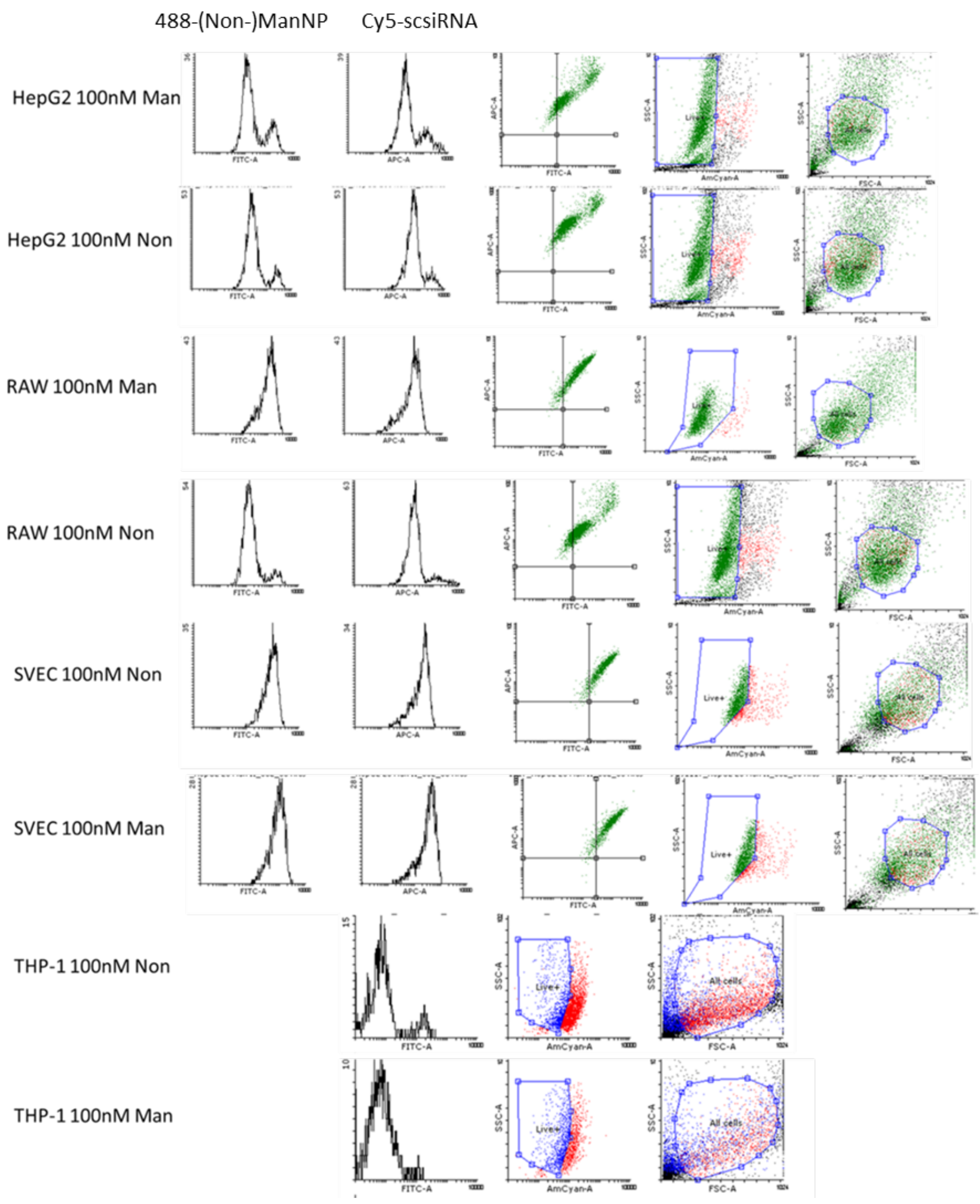

Figure S2. Exemplary dot plots and histograms of the in vitro cellular uptake as determined by FACS analysis: Both Oregon Green labeled (Non-)ManNP (= FITC-A) and Cy5 labelled siRNA (=APC-A) were efficiently taken up in human-hepatocytes (HepG2), -macrophages (THP-1), murine liver endothelial cells (SVEC4-10) and macrophages (RAW).

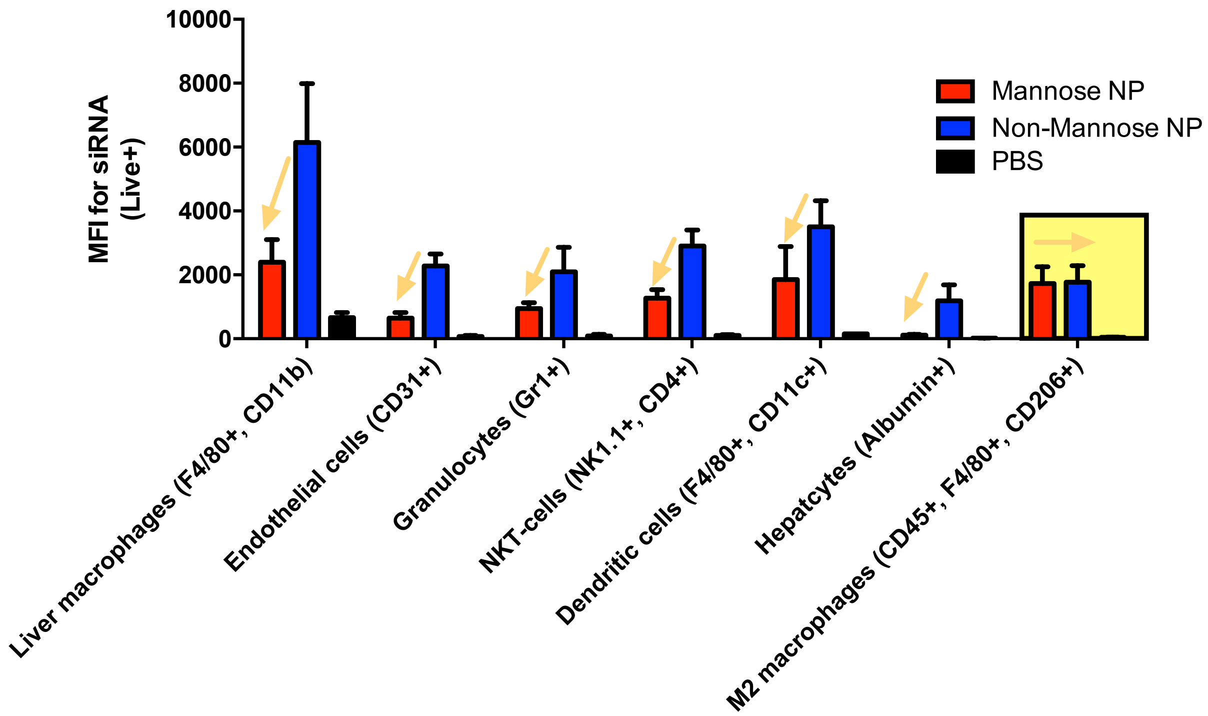

Figure S3. In vivo cellular uptake of RS800-NonNP and RS800-ManNP in (non-)parenchymal liver cells as assessed by FACS analysis of single cell suspension obtained from harvested fibrotic livers. While the non-mannosylated carrier showed a higher overall unspecific uptake in all tested (non-)parenchymal liver cells with no preference for M2 macrophages, uptake of ManNP in CD206+ macrophages remained and increased compared to other cells (indicated by yellow box).

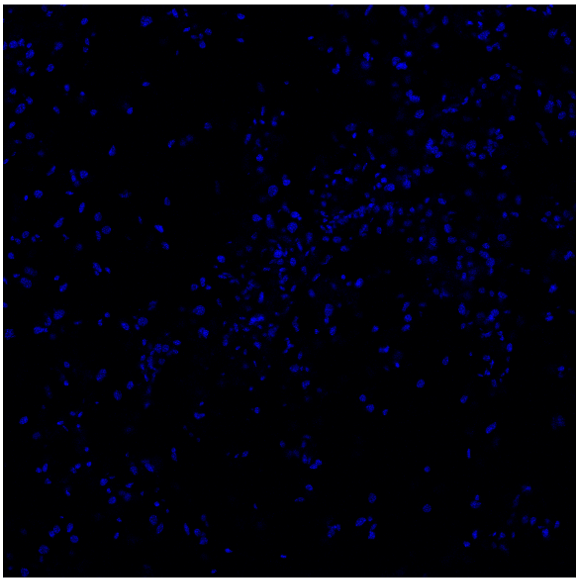

Figure S4. Confocal laser microscopy of a liver cryosection from PBS treated control mice. The control section did not show a fluorescent signal, neither for Cy5-siRNA (red) nor for RS800-(Non-)ManNP.

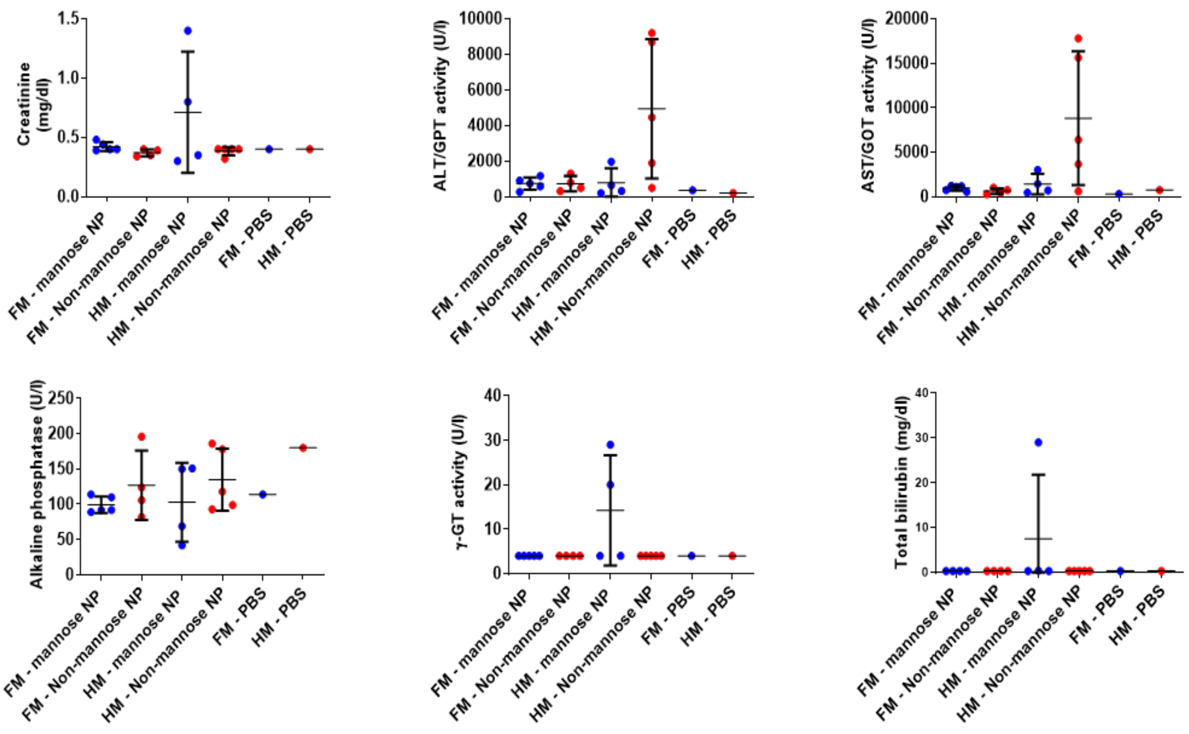

Figure S5. Serum markers of liver and kidney damage from CCl4 liver fibrotic (FM) and healthy (HM) mice treated with Cy5-scsiRNA loaded RS800-(Non-)ManNP (2 mg/kg siRNA) or PBS, respectively. Blood samples were obtained at organ harvest 12 h after i.v. injection of the NP. Normal values and no difference from PBS only injected control mice for liver inflammation (aspartate transaminase, alanine transaminase), cholestasis (alkaline phosphatase, gamma-glutamyltransferase, total bilirubin) and kidney function (creatinine).
